# The Role of Emotion Regulation in Eating Disorders: A Network Meta-Analysis Approach

**DOI:** 10.3389/fpsyt.2022.793094

**Published:** 2022-02-23

**Authors:** Jenni Leppanen, Dalia Brown, Hannah McLinden, Steven Williams, Kate Tchanturia

**Affiliations:** ^1^Department of Neuroimaging, Institute of Psychology, Psychiatry and Neuroscience, King's College London, London, United Kingdom; ^2^School of Education, University of Bristol, Bristol, United Kingdom; ^3^Department of Psychological Medicine, Institute of Psychology, Psychiatry and Neuroscience, King's College London, London, United Kingdom; ^4^South London and Maudsley National Health Service (NHS) Foundation Trust National Eating Disorder Service, London, United Kingdom; ^5^Psychology Department, Illia State University, Tbilisi, Georgia

**Keywords:** eating disorders, emotion regulation, meta-analysis, rumination, acceptance of emotions

## Abstract

**Background:**

Previous theoretical models and reviews have documented a strong connection between emotion dysregulation eating disorder (ED) psychopathology among the general and clinical populations. The aim of this review was to build on this previous work by conducting a network meta-analysis to explore associations between adaptive and maladaptive emotion regulation strategies and ED psychopathology trans-diagnostically across the ED spectrum to identify areas of emotion dysregulation that have the strongest association with symptomatology.

**Methodology:**

A total of 104 studies were included in the meta-analysis and correlation coefficient representing the associations between specific emotion regulation strategies and ED symptomatology were extracted. We ran a Bayesian random effects network meta-analysis and the initial network was well-connected with each emotion regulation strategy being linked to at least one other strategy. We also conducted a network meta-regression to explore whether between-study differences in body mass index (BMI), age, and whether the sample consisted of solely female participants explained any possible network inconsistency.

**Results:**

The network meta-analysis revealed that ruminations and non-acceptance of emotions were most closely associated with ED psychopathology. There was no significant network inconsistency but two comparisons approached significance and thus meta-regressions were conducted. The meta-regressions revealed a significant effect of BMI such that the associations between different emotion regulation strategies and ED symptomatology were weaker among those with low BMI.

**Discussion:**

The present findings build on previous work and highlight the role of rumination and difficulties with accepting emotions as key emotion regulation difficulties in EDs. Additionally, the finding that the associations were weaker among ED patients with low BMI may point toward a complex relationship between ED behaviors and emotion regulation. Taken together, our findings call for interventions that target emotion regulation, specifically rumination and difficulties accepting emotions, in the treatment of EDs.

**Systematic Review Registration:**

https://www.crd.york.ac.uk/prospero/display_record.php?ID=CRD42021249996, PROSPERO, identifier: CRD42021249996.

## Introduction

Emotion regulation refers to strategies used to identify, initiate and modify the course of emotions (1–3). Emotion regulation strategies can be automated or controlled methods used to cope with or modify the external expression and/or internal experience when the emotional state has unwanted impact on a desired outcome (1–3). For example, a young person might suppress their feelings of sadness when moving away from their childhood home in an attempt to not upset their parents thus reducing the external expression of negative emotions. They might then remind themselves they can still visit their parents in an attempt to gain more perspective, reappraise, and reduce the internal experience of sadness. A theoretical framework based on the emotion regulation process model has proposed that emotion regulation strategies can be broadly divided into adaptive and maladaptive strategies (4–7). The adaptive strategies include reappraisal, active problem solving, and mindfulness strategies based on awareness and acceptance of emotions, while the maladaptive strategies include rumination, avoidance, and suppression of emotions (4–6). Although the maladaptive strategies can be useful in some scenarios, this framework focuses on dispositional emotion regulation. Thus, for a given strategy to be considered adaptive is needs to be effective and helpful across contexts (6). Excessive reliance on maladaptive strategies has been found to be linked to anxiety symptoms and to impact treatment response in anxiety disorders (4, 5). Similarly, people with eating disorders (EDs) have been reported to rely more on maladaptive than adaptive emotion regulation strategies (8–10). Thus, this review will focus on further investigating the associations between different adaptive and maladaptive emotion regulation strategies an ED psychopathology.

Over the years there has been a great deal of interest in exploring emotion regulation within EDs; it has been proposed that difficulties in emotion regulation is one of the social-emotional factors contributing to the development and maintenance of disordered eating (9, 11–13). Large scale systematic reviews and meta-analyses have reported links between ED symptomatology and dispositional tendency toward maladaptive emotion regulation and emotion dysregulation among people with anorexia nervosa (AN), bulimia nervosa (BN), and binge eating disorder (BED), both when assessed under laboratory conditions and under naturalistic conditions in studies using ecological momentary assessments (EMAs) (9, 11, 13). Recently a meta-analytic review by Prefit et al. (12) examined correlations between the use of various emotion regulation strategies and ED psychopathology across all ED diagnoses. The authors focused on examining individual adaptive strategies, including emotional awareness, clarity about emotions, acceptance of emotions, cognitive reappraisal, and problem solving, as well as maladaptive strategies, such as avoidance, rumination and suppression of emotions. The findings showed that across the board maladaptive emotion regulation strategies were positively associated with ED symptoms while the use of adaptive strategies was negatively associated with ED psychopathology. Interestingly, this was the case across ED diagnoses and the authors found no significant evidence of group differences in the analyses. Additionally, no particular strategy came out as more closely associated with ED psychopathology, instead it appeared that all adaptive strategies were equally negatively associated and all maladaptive strategies were equally positively associated with ED symptomatology.

Although the findings from previous reviews highlight the interesting associations between emotion regulation difficulties and ED psychopathology, whether there is a particular aspect of adaptive or maladaptive emotion regulation that has a most important role in EDs remains unclear. This question could be approached using the network meta-analysis technique, which explores comparative associations of several emotion regulation strategies by utilizing existing direct evidence to estimate indirect evidence. This approach has been previously used to compare different interventions to identify most effective treatments (14–17). More recently the network meta-analysis approach has also been used to compare associations in the field of educations (18). The authors demonstrated a method of applying network meta-analysis to investigate which test was the most closely associated with pupils reading fluency, in an attempt to identify the most useful reading test to be used in school setting. Such a methods could also be applied to emotion regulation in EDs to identify if there is a particular strategy that is most closely associated with ED symptomatology. This approach could inform the development of novel interventions for EDs rather than aiming to generally increase the use of adaptive strategies and reduce reliance on maladaptive strategies.

The aim of this meta-analytic review was to build on and synthesize previous literature by comparing findings from previous work examining the association between different aspects of adaptive and maladaptive emotion regulation and ED psychopathology among people with a diagnosis of an ED. Thus, this review aimed to build on the recent work by Prefit et al. (12) and conduct a network meta-analysis. We compared the use of adaptive emotion regulation strategies, including acceptance of emotions, awareness of emotions, problem solving, and cognitive reappraisal, as well as maladaptive emotion regulation strategies, such as avoidance, rumination, and emotion suppression. The objective was to identify aspects of emotion regulation that have closest association with ED symptomatology to identify useful targets for therapeutic interventions. As previous work has suggested that difficulties in emotion regulation may be trans-diagnostic (12, 13), we assessed the associations between the use of different emotion regulation strategies and ED symptomatology across ED diagnoses. Because findings from previous meta-analyses have reported very similar associations between different emotion regulation strategies and ED symptomatology, we did not have *a priori* expectations regarding which one or more emotion regulation strategies would emerge as being most closely associated with ED psychopathology.

## Materials and Methods

### Literature Search

The following keywords were used to search electronic databases including PsychINFO, PsychARTICLES, Medline, Scopus, Pubmed, and Web of Knowledge: *(“eating disorder” OR “anorexia nervosa” OR “bulimia nervosa” OR “binge eating disorder”) AND (“emotion regulation” OR “emotion dysregulation” OR “affect regulation” OR “affect dysregulation” OR “avoidance” OR “suppression” OR “rumination” OR “problem solving” OR “problem coping” OR “awareness” OR “mindfulness” OR “acceptance” OR “clarity” OR “appraisal” OR “reappraisal”)*. Where possible filters were used to exclude animal studies, case reports, reviews, and studies not published in English. The initial search was conducted in April 2021 and was updated in August 2021. Bibliographies of included papers and the previous review by Prefit et al. (12) were searched to identify any studies missed in the initial search.

### Inclusion Criteria

To be included in the review the studies were requires to meet the following inclusion criteria: (1) include a group of adults or adolescents [10–19 years old (19)] with a diagnosis of an ED, (2) measure some aspect of emotion regulation, (3) measure ED symptomatology, (4) be published in English, and (5) be published in a peer reviewed journal. In the case of longitudinal or interventional studies, only emotion regulation and ED symptomatology measures taken at baseline or pre-treatment were included. Additionally, studies that only assessed emotion regulation and/or ED symptomatology at a later stage, post-intervention or at treatment follow-up, were excluded. As we were interested in comparing different emotion regulation strategies, any studies that reported only overall measures and did not assess a specific aspects of emotion regulation were not included. Studies that instructed participants to use specific emotion regulation strategies or assessed regulation of eating or other aspects of food intake, or emotion regulation related behaviors that were to do with food, eating or bodies, such as food avoidance, or body image acceptance, were also excluded. Finally, studies that included young children or clinical populations with diagnoses other than an ED were excluded.

### Study Selection

**Figure 1:**
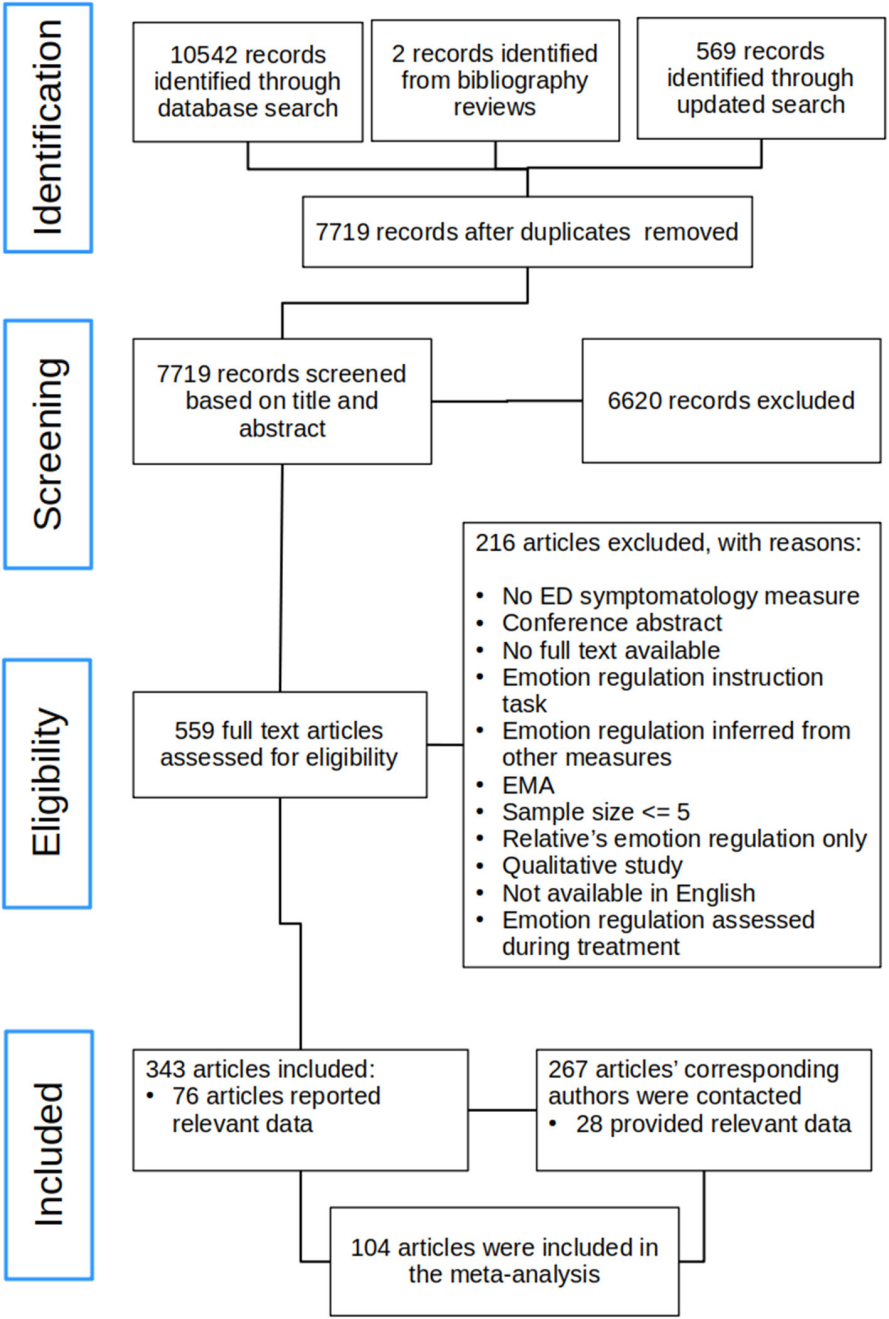
Flow diagram. ED, Eating disorder; EMA, ecological momentary assessment.

This review was conducted in accordance with the PRISMA guidelines and the flow diagram detailing the literature search and study selection is presented in Figure 1. The initial search and screening based on title and abstract was conducted by J.L along with H.M. The included full text articles were then assessed for eligibility and decisions about final inclusion or exclusion of articles was made initially by the first author. All excluded papers were screened again based on title and abstract by H.M. to ensure no relevant studies were missed. In case uncertainty regarding whether a given study should be included or excluded, the full text article was brought to the rest of the team for consensus meeting and team discussion.

### Data Collection and Synthesis

To conduct the network meta-analysis, we took a similar approach to a recent review investigating associations between oral reading fluency and text complexity measures to identify an assessment method most closely correlated with reading fluency (18). Thus, correlation coefficient between an emotion regulation measure and ED symptomatology was extracted from all included papers along with the ED group sample size, the aspect of emotion regulation assessed, and the type of ED symptomatology measure used. Only correlation analysis conducted within the ED group were included. If only correlations for the whole sample which included healthy participants or participants with other diagnoses were reported, the corresponding author was contacted to obtain access to a within ED group correlation coefficient. If a study included more than one ED group separate correlation coefficients for each diagnostic group or one correlation coefficient for the whole mixed ED group were included depending on what was reported. We did not favor separate correlation coefficients as the meta-analysis included all ED diagnoses. The relevant correlation coefficients were not reported in the main text or Supplementary Materials of 267 studies and the corresponding authors were contacted by D.B. to gain access to the data. Correlation coefficients which were not reported in 28 of the included manuscripts, were obtained through personal correspondence. Additional data regarding the age of the ED group, body mass index (BMI), and percentage of female participants was also extracted where available.

### Methods Used to Assess Emotion Regulation

We included studies that assessed the use of adaptive emotion regulation strategies, including reappraisal, acceptance of emotions, emotional awareness, and problem solving, as well as the use of maladaptive emotion regulation strategies, such as avoidance, suppression, and rumination (4–7).

#### Adaptive Emotion Regulation Measures

Thirty-one studies assessed the association between acceptance of emotions and ED symptomatology. Four different methods were used to assess acceptance of emotions (see Table 1 for details), all of which were self-report questionnaire measures. The most commonly used measure was the Difficulties in Emotion Regulation Scale [DERS; (123)] non-acceptance subscale (*N* = 24). For the purposes of the meta-analysis all correlation coefficients had to have the same direction, with positive correlations indicating that a greater level of ED symptomatology was associated with more difficulties in emotion regulation. Therefore, the correlations that included measures of acceptance of emotions were reversed, while correlation that included measures of non-acceptance or difficulties in accepting emotions were not reversed. This was done to reflect reverse scoring of adaptive emotion regulation, whereby higher scores indicate less acceptance and, thus, this review assessed associations between non-acceptance of emotions and ED symptomatology.

**Table 1:** Study characteristics.

**Study**	**ED sample**	**Sample size**	**N (%) Female**	**Age M (SD)**	**BMI M (SD)**	**Emotion regulation measure**	**ED symptomatology measure**	**Emotion regulation category**	**Correlation [95% CI]**
**Acceptance**									
Brytek-Matera and Schiltz (20)	AN, BN	52	52 (100%)	19.6 (2.6)	18.1 (2.5)	SVF120 Acceptance	ED pathology	Non-acceptance	0.56 [0.28, 0.84]
Aloi et al. (21)	BED	46	40 (87%)	40.6 (12.7)	38.8 (7.1)	DERS non-acceptance	BES total	Non-acceptance	0.32 [0.02, 0.62]
Aloi et al. (22)	BED	155	86.5%	41.2 (13.2)	37.9 (10.4)	DERS non-acceptance	EDI total	Non-acceptance	0.16 [0.0004, 0.32]
Blomquist et al. (23)	BED	168	120 (71.4%)	48.3 (10.2)	38.8 (5.7)	DERS non-acceptance	ELOC total	Non-acceptance	0.40 [0.25, 0.56]
Bodell et al. (24)	BN, BED, OSFED	97	89 (91.8%)	38.4 (13.9)	37.2 (11.3)	DERS non-acceptance	EDEQ total	Non-acceptance	0.41 [0.20, 0.61]
Brown et al. (25)	AN, BN, ARFID, OSFED	364	94.4%	20.9 (9.3)	NR	DERS non-acceptance	EDEQ total	Non-acceptance	0.59 [0.49, 0.69]
Hazzard et al. (26)	BED	112	92 (82.1%)	39.7 (13.4)	> 21.0	DERS non-acceptance	EDE total	Non-acceptance	0.23 [0.05, 0.42]
Juarascio et al. (27)	AN, BN, ENDOS	120	120 (100%)	26.7 (9.2)	NR	DERS non-acceptance	EDQOL total	Non-acceptance	0.23 [0.05, 0.42]
Kenny et al. (28)	BED	71	93%	40.4 (11.4)	37.7 (9.6)	DERS non-acceptance	EDEQ total	Non-acceptance	0.47 [0.23, 0.71]
Lavender et al. (29)	BN	80	72 (90%)	27.3 (9.6)	23.9 (5.5)	DERS non-acceptance	EDE total	Non-acceptance	0.31 [0.09, 0.53]
MacDonald et al. (30)	BN, OSFED	104	93.3%	28.8 (9.0)	24.1 (6.3)	DERS non-acceptance	EDEQ total	Non-acceptance	0.37 [0.17, 0.56]
MacDonald et al. (31)	BN, PD	44	44 (100%)	27.3 (8.4)	24.6 (5.8)	DERS non-acceptance	EDEQ total	Non-acceptance	0.29 [-0.02, 0.59]
Michael and Juarascio (32)	AN, BN, BED	111	111 (100%)	26.7 (9.2)	NR	DERS non-acceptance	EDEQ total	Non-acceptance	0.35 [0.26, 0.54]
Monell et al. (33)	AN, BN, BED, OSFED	999	999 (100%)	24.8 (8.4)	21.5 (5.2)	DERS non-acceptance	EDEQ total	Non-acceptance	0.36 [0.30, 0.43]
Pisetsky et al. (35)	AN, BN, BED, OSFED	110	93.6%	33.5 (12.2)	28.1 (11.2)	DERS non-acceptance	EDEQ total	Non-acceptance	0.22 [0.03, 0.41]
Racine and Wildes (36)	AN	192	183 (95.3%)	26.5 (10.2)	15.7 (1.8)	DERS non-acceptance	EDE total	Non-acceptance	0.38 [0.25, 0.53]
Racine and Wildes (37)	AN	191	182 (95.3%)	26.5 (10.2)	15.7 (1.8)	DERS non-acceptance	EDE total	Non-acceptance	0.89 [0.74, 1.03]
Rania et al. (38)	AN, BN, BED, OSFED	2405	2303 (95.8%)	22.5 (8.6)	21.7 (6.1)	DERS non-acceptance	EDEQ total	Non-acceptance	0.38 [0.34, 0.42]
Southward et al. (39)	BN, BED	107	107 (100%)	35.2 (12.5)	30.7 (11.1)	DERS non-acceptance	EDE total	Non-acceptance	0.37 [0.17, 0.56]
Steinglass et al. (40)	AN	20	20 (100%)	32.0 (10.4)	15.8 (1.6)	DERS non-acceptance	EDE restraint	Non-acceptance	1.00 [0.52, 1.47]
Svaldi et al. (41)	AN, BN, BED	63	63 (100%)	30.7 (8.6)	25.4 (4.3)	DERS non-acceptance	EDEQ total	Non-acceptance	0.50 [0.24, 0.75]
Turan et al. (42)	BED	32	18 (57.6%)	15.0 (1.4)	34.6 (3.0)	DERS non-acceptance	EDEQ total	Non-acceptance	0.22 [-0.15, 0.58]
Wisting et al. (43)	AN, BN, BED	272	272 (100%)	29.4 (8.8)	24.3 (8.4)	DERS non-acceptance	EDEQ total	Non-acceptance	0.47 [0.35, 0.59]
Wolz et al. (44)	AN, BN, BED, OSFED	134	121 (90.3%)	28.8 (10.4)	25.0 (9.1)	DERS non-acceptance	EDI total	Non-acceptance	0.54 [0.36, 0.71]
Wyssen et al. (45)	AN, BN	112	112 (100%)	23.0 (4.3)	19.8 (2.1)	DERS non-acceptance	EDEQ total	Non-acceptance	0.42 [0.24, 0.61]
Cowdrey and Park (46)	AN	42	42 (100%)	24.0 (8.3)	19.6 (2.5)	AAQ-II total	EDEQ total	Non-acceptance	0.51 [0.20, 0.82]
Dajon and Sudres (47)	AN, BN, BED, OSFED	97	97 (100%)	35.8 (13.1)	24.7 (7.0)	AAQ-II total	EDI drive for thinness	Non-acceptance	−0.27 [-0.47,−0.07]
Lee et al. (48)	AN, BN, EDNOS	132	132 (100%)	19.1 (5.8)	19.3 (3.7)	AAQ-II total	EDI drive for thinness	Non-acceptance	0.63 [0.46, 0.81]
Manwaring et al. (49)	AN, BN, EDNOS	281	264 (94.0%)	26.6 (9.4)	17.3 (3.7)	AAQ-W total	EDI ED risk	Non-acceptance	0.99 [0.88, 1.11]
Walden et al. (50)	AN, BN, EDNOS	617	(94.6%)	28.0 (10.0)	NR	AAQ-W total	EDI drive for thinness	Non-acceptance	0.81 [0.73, 0.89]
Butryn et al. (51)	AN, BN, EDNOS	88	88 (100%)	26.5 (12.2)	NR	PHLMS acceptance	EDEQ total	Non-acceptance	0.26 [0.04, 0.47]
**Awareness**									
Aloi et al. (21 )	BED	46	40 (87%)	40.6 (12.7)	38.8 (7.1)	DERS lack of awareness	BES total	Lack of awareness	0.09 [-0.21, 0.39]
Aloi et al. (22)	BED	155	86.5%	41.2 (13.2)	37.9 (10.4)	DERS lack of awareness	EDI total	Lack of awareness	0.12 [-0.04, 0.28]
Blomquist et al. (23)	BED	168	120 (71.4%)	48.3 (10.2)	38.8 (5.7)	DERS lack of awareness	ELOC total	Lack of awareness	0.04 [-0.12, 0.19]
Bodell et al. (24)	BN, BED, OSFED	97	89 (91.8%)	38.4 (13.9)	37.2 (11.3)	DERS lack of awareness	EDEQ total	Lack of awareness	−0.08 [-0.28, 0.12]
Brown et al. (25)	AN, BN, ARFID, OSFED	364	94.4%	20.9 (9.3)	NR	DERS lack of awareness	EDEQ total	Lack of awareness	0.35 [0.25, 0.45]
Hazzard et al. (26)	BED	112	92 (82.1%)	39.7 (13.4)	> 21.0	DERS lack of awareness	EDE total	Lack of awareness	0.07 [-0.12, 0.26]
Juarascio et al. (27)	AN, BN, ENDOS	120	120 (100%)	26.7 (9.2)	NR	DERS lack of awareness	EDQOL total	Lack of awareness	−0.07 [-0.25, 1.11]
Kenny et al. (28)	BED	71	93%	40.4 (11.4)	37.7 (9.6)	DERS lack of awareness	EDEQ total	Lack of awareness	−0.06 [-0.30, 0.18]
Lavender et al. (29)	BN	80	72 (90%)	27.3 (9.6)	23.9 (5.5)	DERS lack of awareness	EDE total	Lack of awareness	0.04 [-0.18, 0.26]
MacDonald et al. (30)	BN, OSFED	104	93.3%	28.8 (9.0)	24.1 (6.3)	DERS lack of awareness	EDEQ total	Lack of awareness	0.14 [-0.05, 0.34]
MacDonald et al. (31)	BN, PD	44	44 (100%)	27.3 (8.4)	24.6 (5.8)	DERS lack of awareness	EDEQ total	Lack of awareness	0.31 [0.003, 0.62]
Michael and Juarascio (32)	AN, BN, BED	109	109 (100%)	26.7 (9.2)	NR	DERS lack of awareness	EDEQ total	Lack of awareness	−0.31 [-0.50,−0.12]
Monell et al. (33)	AN, BN, BED, OSFED	999	999 (100%)	24.8 (8.4)	21.5 (5.2)	DERS lack of awareness	EDEQ total	Lack of awareness	0.30 [0.24, 0.36]
Pisetsky et al. (35)	AN, BN, BED, OSFED	110	93.6%	33.5 (12.2)	28.1 (11.2)	DERS lack of awareness	EDEQ total	Lack of awareness	0.32 [0.13, 0.51]
Racine and Wildes (36)	AN	192	183 (95.3%)	26.5 (10.2)	15.7 (1.8)	DERS lack of awareness	EDE total	Lack of awareness	0.38 [0.25, 0.53]
Racine and Wildes (37)	AN	191	182 (95.3%)	26.5 (10.2)	15.7 (1.8)	DERS lack of awareness	EDE total	Lack of awareness	0.55 [0.41, 0.69]
Rania et al. (38)	AN, BN, BED, OSFED	2405	2303 (95.8%)	22.5 (8.6)	21.7 (6.1)	DERS lack of awareness	EDEQ total	Lack of awareness	0.28 [0.24, 0.32]
Southward et al. (39)	BN, BED	107	107 (100%)	35.2 (12.5)	30.7 (11.1)	DERS lack of awareness	EDE total	Lack of awareness	0.05 [-0.14, 0.24]
Steinglass et al. (40)	AN	20	20 (100%)	32.0 (10.4)	15.8 (1.6)	DERS lack of awareness	EDE restraint	Lack of awareness	0.16 [-0.31, 0.64]
Svaldi et al. (41)	AN, BN, BED	63	63 (100%)	30.7 (8.6)	25.4 (4.3)	DERS lack of awareness	EDEQ total	Lack of awareness	0.34 [0.09, 0.60]
Turan et al. (42)	BED	32	18 (57.6%)	15.0 (1.4)	34.6 (3.0)	DERS lack of awareness	EDEQ total	Lack of awareness	0.14 [-0.22, 0.51]
Wisting et al. (43)	AN, BN, BED	272	272 (100%)	29.4 (8.8)	24.3 (8.4)	DERS lack of awareness	EDEQ total	Lack of awareness	0.30 [0.18, 0.42]
Wolz et al. (44)	AN, BN, BED, OSFED	134	121 (90.3%)	28.8 (10.4)	25.0 (9.1)	DERS lack of awareness	EDI total	Lack of awareness	0.15 [-0.02, 0.32]
Wyssen et al. (45)	AN, BN	112	112 (100%)	23.0 (4.3)	19.8 (2.1)	DERS lack of awareness	EDEQ total	Lack of awareness	0.32 [0.13, 0.51]
Legenbauer et al. (52)	BN	20	20 (100%)	22.7 (4.4)	20.8 (2.3)	ACF total	EDI drive for thinness	Lack of awareness	1.39 [0.91, 1.86]
Bernatova and Svetlak (53)	AN, BN	73	73 (100%)	15.5 (1.3)	17.3 (3.4)	LEAS awareness of emotions in self	RS total	Lack of awareness	−0.40 [-0.63,−0.17]
Butryn et al. (51)	AN, BN, EDNOS	88	88 (100%)	26.5 (12.2)	NR	PHLMS awareness	EDEQ total	Lack of awareness	0.50 [0.28, 0.71]
Dunne et al. (54)	AN, atypical AN	59	59 (100%)	25.7 (8.8)	19.0	CAMS-R total	EDEQ total	Lack of awareness	0.25 [-0.01, 0.51]
Torres et al. (55)	BED	7	7 (100%)	38.3 (9.3)	34.6 (3.9)	EPS impoverished emotion experience	DEBQ total	Lack of awareness	0.40 [-0.58, 1.38]
Messer et al. (56)	BN	145	NR	NR	NR	MAAS total	EDEQ total	Lack of awareness	0.12 [-0.04, 0.29]
	BED	150	NR	NR	NR	MAAS total	EDEQ total	Lack of awareness	0.18 [0.02, 0.34]
Compare et al. (57)	BED	150	98 (65.3%)	49.3 (4.1)	33.1 (1.2)	FFMQ total	BES total	Lack of awareness	0.39 [0.23, 0.55]
Cowdrey and Park (46)	AN	42	42 (100%)	24.0 (8.3)	19.6 (2.5)	FFMQ total	EDEQ total	Lack of awareness	0.68 [0.36, 0.99]
Lattimore et al. (58)	AN, BN, BED	39	39 (100%)	29.0 (9.4)	NR	FFMQ total	EDI drive for thinness	Lack of awareness	0.48 [0.16, 0.81]
Pepping et al. (59)	BN, BED, EDNOS	55	55 (100%)	39.0 (12.7)	NR	FFMQ total	EDI dive for thinness	Lack of awareness	0.22 [-0.05, 0.50]
Pinto-Gouveia et al. (60)	BED	33	33 (100%)	41.9 (9.8)	34.8 (5.2)	FFMQ total	EDE total	Lack of awareness	−0.07 [-0.43, 0.28]
Espel-Huynh et al. (61)	AN, BN, EDNOS	531	531 (100%)	25.3 (11.1)	NR	SMQ total	PMT ED behaviours	Lack of awareness	0.42 [0.34, 0.51]
Scharff et al. (62)	AN, BN, OSFED	1042	1042 (100%)	25.3 (10.6)	23.3 (8.9)	SMQ total	EDEQ total	Lack of awareness	0.47 [0.41, 0.53]
Manwaring et al. (49)	AN, BN, EDNOS	281	264 (94.0%)	26.6 (9.4)	17.3 (3.7)	KIMS total	EDI ED risk	Lack of awareness	0.99 [0.88, 1.11]
Problem solving
Paterson et al. (63)	AN	55	55 (100%)	24.6 (6.8)	14.6 (2.2)	SPSI-R negative problem orientation	EDEQ total	Problem solving difficulties	0.57 [0.29, 0.84]
Paterson et al. (64)	AN	27	27 (100%)	26.5 (7.9)	18.0 (3.3)	SPSI-R negative problem orientation	EAT total	Problem solving difficulties	0.44 [0.04, 0.84]
Sternheim et al. (65)	AN	30	30 (100%)	24.0 (6.4)	16.0 (2.1)	SPSI-R negative problem orientation	EDEQ total	Problem solving difficulties	0.0001 [-0.38, 0.38]
Brytek-Matera and Schiltz (20)	AN, BN	52	52 (100%)	19.6 (2.6)	18.1 (2.5)	COPE	ED pathology	Problem solving difficulties	0.47 [0.19, 0.75]
Fitzsimmons and Bardone-Cone (66)	AN, BN, EDNOS	151	151 (100%)	23.6 (4.7)	NR	CISS task-oriented coping	EAT total	Problem solving difficulties	0.24 [0.08, 0.41]
Marchiol et al. (67)	AN	34	34 (100%)	25.7 (10.6)	NR	CISS task-oriented coping	EDI ED risk	Problem solving difficulties	0.03 [-0.33, 0.38]
	BN	30	30 (100%)	24.0 (8.4)	NR	CISS task-oriented coping	EDI ED risk	Problem solving difficulties	0.34 [-0.04, 0.72]
	BED	29	29 (100%)	37.5 (12.7)	NR	CISS task-oriented coping	EDI ED risk	Problem solving difficulties	0.14 [-0.24, 0.53]
Spoor et al. (68)	AN, BN, BD, EDNOS	125	125 (100%)	29.9 ( 9.2)	NR	CISS task-oriented coping	DEBQ emotional eating	Problem solving difficulties	0.14 [-0.04, 0.32]
Nagata et al. (34)	AN, BN	161	161 (100%)	22.8 (4.6)	16.5 (2.6)	CISS task-oriented coping	EDI drive for thinness	Problem solving difficulties	0.05 [-0.011, 0.21]
Aloi et al. (21)	BED	46	40 (87%)	40.6 (12.7)	38.8 (7.1)	DERS difficulties in goal directed behaviour	BES total	Problem solving difficulties	0.32 [0.03, 0.62]
Aloi et al. (22)	BED	155	86.5%	41.2 (13.2)	37.9 (10.4)	DERS difficulties in goal directed behaviour	EDI total	Problem solving difficulties	0.10 [-0.06, 0.26]
Blomquist et al. (23)	BED	168	120 (71.4%)	48.3 (10.2)	38.8 (5.7)	DERS difficulties in goal directed behaviour	ELOC total	Problem solving difficulties	0.41 [0.25, 0.56]
Bodell et al. (24)	BN, BED, OSFED	97	89 (91.8%)	38.4 (13.9)	37.2 (11.3)	DERS difficulties in goal directed behaviour	EDEQ total	Problem solving difficulties	0.15 [-0.10, 0.35]
Brown et al. (25)	AN, BN, ARFID, OSFED	364	94.4%	20.9 (9.3)	NR	DERS difficulties in goal directed behaviour	EDEQ total	Problem solving difficulties	0.40 [0.29, 0.50]
Hazzard et al. (26)	BED	112	92 (82.1%)	39.7 (13.4)	> 21.0	DERS difficulties in goal directed behaviour	EDE total	Problem solving difficulties	0.21 [0.02, 0.40]
Juarascio et al. (27)	AN, BN, ENDOS	120	120 (100%)	26.7 (9.2)	NR	DERS difficulties in goal directed behaviour	EDQOL total	Problem solving difficulties	0.21 [0.03, 0.39]
Kenny et al. (28)	BED	71	93%	40.4 (11.4)	37.7 (9.6)	DERS difficulties in goal directed behaviour	EDEQ total	Problem solving difficulties	0.31 [0.07, 0.55]
Lavender et al. (29)	BN	80	72 (90%)	27.3 (9.6)	23.9 (5.5)	DERS difficulties in goal directed behaviour	EDE total	Problem solving difficulties	0.27 [0.04, 0.49]
MacDonald et al. (30)	BN, OSFED	104	93.3%	28.8 (9.0)	24.1 (6.3)	DERS difficulties in goal directed behaviour	EDEQ total	Problem solving difficulties	0.35 [0.16, 0.55]
MacDonald et al. (31)	BN, PD	44	44 (100%)	27.3 (8.4)	24.6 (5.8)	DERS difficulties in goal directed behaviour	EDEQ total	Problem solving difficulties	0.17 [-0.13, 0.48]
Michael and Juarascio (32)	AN, BN, BED	111	111 (100%)	26.7 (9.2)	NR	DERS difficulties in goal directed behaviour	EDEQ total	Problem solving difficulties	0.32 [0.13, 0.51]
Monell et al. (33)	AN, BN, BED, OSFED	999	999 (100%)	24.8 (8.4)	21.5 (5.2)	DERS difficulties in goal directed behaviour	EDEQ total	Problem solving difficulties	0.24 [0.18, 0.30]
Pisetsky et al. (35)	AN, BN, BED, OSFED	110	93.6%	33.5 (12.2)	28.1 (11.2)	DERS difficulties in goal directed behaviour	EDEQ total	Problem solving difficulties	0.20 [0.01, 0.39]
Racine and Wildes (36)	AN	192	183 (95.3%)	26.5 (10.2)	15.7 (1.8)	DERS difficulties in goal directed behaviour	EDE total	Problem solving difficulties	0.19 [0.05, 0.33]
Racine and Wildes (37)	AN	191	182 (95.3%)	26.5 (10.2)	15.7 (1.8)	DERS difficulties in goal directed behaviour	EDE total	Problem solving difficulties	0.78 [0.63, 0.92]
Rania et al. (38)	AN, BN, BED, OSFED	2405	2303 (95.8%)	22.5 (8.6)	21.7 (6.1)	DERS difficulties in goal directed behaviour	EDEQ total	Problem solving difficulties	0.33 [0.29, 0.37]
Southward et al. (39)	BN, BED	107	107 (100%)	35.2 (12.5)	30.7 (11.1)	DERS difficulties in goal directed behaviour	EDE total	Problem solving difficulties	0.30 [0.11, 0.49]
Steinglass et al. (40)	AN	20	20 (100%)	32.0 (10.4)	15.8 (1.6)	DERS difficulties in goal directed behaviour	EDE restraint	Problem solving difficulties	0.51 [0.04, 0.99]
Svaldi et al. (41)	AN, BN, BED	63	63 (100%)	30.7 (8.6)	25.4 (4.3)	DERS difficulties in goal directed behaviour	EDEQ total	Problem solving difficulties	0.50 [0.24, 0.75]
Turan et al. (42)	BED	32	18 (57.6%)	15.0 (1.4)	34.6 (3.0)	DERS difficulties in goal directed behaviour	EDEQ total	Problem solving difficulties	0.01 [-0.35, 0.38]
Wisting et al. (43)	AN, BN, BED	272	272 (100%)	29.4 (8.8)	24.3 (8.4)	DERS difficulties in goal directed behaviour	EDEQ total	Problem solving difficulties	0.27 [0.15, 0.39]
Wolz et al. (44)	AN, BN, BED, OSFED	134	121 (90.3%)	28.8 (10.4)	25.0 (9.1)	DERS difficulties in goal directed behaviour	EDI total	Problem solving difficulties	0.54 [0.36, 0.71]
Wyssen et al. (45)	AN, BN	112	112 (100%)	23.0 (4.3)	19.8 (2.1)	DERS difficulties in goal directed behaviour	EDEQ total	Problem solving difficulties	0.35 [0.16, 0.53]
Svaldi et al. (69)	BED	25	25 (100%)	42.8 (10.1)	29.5 (3.9)	MEPS effectiveness	DEBQ total	Problem solving difficulties	0.29 [-0.13, 0.71]
Davies et al. (70)	AN, BN, BED, EDNOS	92	92 (100%)	30.2 (9.3)	21.8 (4.0)	UCL active problem solving by reassuring thoughts	EDI drive for thinness	Problem solving difficulties	0.11 [-0.10, 0.32]
**Reappraisal**									
Corstorphine et al. (71)	AN, BN, EDNOS	72	72 (100%)	24.5 (7.8)	22.7 (8.7)	DTS accept and manage	EDI drive for thinness	Lack of cognitive reappraisal	0.23 [-0.003, 0.47]
Oldershaw et al. (72)	AN, EDNOS	40	37 (92.5%)	26.0 (8.8)	16.6 (1.3)	DTS accept and manage	EDEQ total	Lack of cognitive reappraisal	0.06 [-0.27, 0.38]
Raykos et al. (73)	AN, BN, EDNOS	204	204 (100%)	25.8 (9.7)	19.5 (3.1)	DTS accept and manage	EDEQ total	Lack of cognitive reappraisal	−0.11 [-0.25, 0.03]
Danner et al. (74)	AN restrictive	20	20 (100%)	21.1 (3.2)	17.6 (2.2)	ERQ reappraisal	EDDS symptomatology	Lack of cognitive reappraisal	0.04 [-0.44, 0.52]
	AN binge-purge, BN	30	30 (100%)	21.7 (2.3)	19.8 (2.2)	ERQ reappraisal	EDDS total	Lack of cognitive reappraisal	0.34 [-0.03, 0.72]
Danner et al. (75)	AN, BN, BED, EDNOS	123	123 (100%)	28.6 (8.4)	23.8 (3.3)	ERQ reappraisal	EDDS total	Lack of cognitive reappraisal	0.15 [-0.03, 0.33]
Davies et al. (76)	AN, BN	103	103 (100%)	26.4 (8.5)	18.1 (4.1)	ERQ reappraisal	EDEQ total	Lack of cognitive reappraisal	0.29 [0.10, 0.49]
Rothschild-Yakar et al. (77)	AN, BN	25	25 (100%)	17.2 (2.8)	20.4 (4.0)	ERQ reappraisal	EAT total	Lack of cognitive reappraisal	0.28 [-0.14, 0.69]
Svaldi et al. (41)	AN, BN, BED	63	63 (100%)	30.7 (8.6)	25.4 (4.3)	ERQ reappraisal	EDEQ total	Lack of cognitive reappraisal	0.24 [-0.01, 0.50]
**Avoidance**									
Butryn et al. (51 )	AN, BN, EDNOS	88	88 (100%)	26.5 (12.2)	NR	EAQ avoidance of positive emotions	EDEQ total	Avoidance	0.41 [0.20, 0.62]
Wildes et al. (78)	AN	75	74 (98.7%)	26.3 (8.6)	15.8 (1.8)	EAQ total	EDI drive for thinness	Avoidance	0.39 [0.16, 0.62]
Espel-Huynh et al. (61)	AN, BN, EDNOS	531	531 (100%)	25.3 (11.1)	NR	BEAQ total	PMT ED behaviours	Avoidance	0.26 [0.17, 0.34]
Espel-Huynh et al. (61)	AN, BN, BED, OSFED	625	625 (100%)	25.1 (10.7)	NR	BEAQ total	EDEQ total	Avoidance	0.42 [0.35, 0.50]
Scharff et al. (62)	AN, BN, OSFED	1042	1042 (100%)	25.3 (10.6)	23.3 (8.9)	BEAQ total	EDEQ total	Avoidance	0.37 [0.31, 0.43]
Waller and Kyriacou Marcoulides (79)	AN, BN	102	102 (100%)	26.3 (7.7)	16.4 (1.6)	BSBS avoidance	EDEQ total	Avoidance	0.29 [0.10, 0.49]
Paterson et al. (63)	AN	55	55 (100%)	24.6 (6.8)	14.6 (2.2)	SPSI-R avoidance style	EDEQ total	Avoidance	0.35 [0.07, 0.62]
Paterson et al. (64)	AN	27	27 (100%)	26.5 (7.9)	18.0 (3.3)	SPSI-R avoidance style	EAT total	Avoidance	0.47 [0.07, 0.87]
Sternheim et al. (65)	AN	30	30 (100%)	24.0 (6.4)	16.0 (2.1)	SPSI-R avoidance style	EDEQ total	Avoidance	0.0001 [-0.38, 0.38]
Mason et al. (80)	BN, sub-threshold BN	204	204 (100%)	25.7 (8.9)	23.0 (5.3)	DAPP-BQ social avoidance	EEQ total	Avoidance	0.38 [0.24, 0.52]
Thaler et al. (81)	AN	55	55 (100%)	23.5 (5.8)	15.2 (5.5)	DAPP-BQ social avoidance	EDEQ total	Avoidance	0.16 [-0.11, 0.43]
Cockell et al. (82)	AN, sub-threshold AN	80	80 (100%)	25.3 (8.5)	17.3 (2.3)	DB functional avoidance	EDI total	Avoidance	0.06 [-0.16, 0.28]
Delinsky et al. (83)	AN, BN, EDNOS	67	67 (100%)	18.9 (range = 16.0 – 23.0)	NR	DB functional avoidance	EDEQ total	Avoidance	0.37 [0.12, 0.61]
Fitzsimmons and Bardone-Cone (66)	AN, BN, EDNOS	151	151 (100%)	23.6 (4.7)	NR	CISS avoidance distraction	EAT-26 total	Avoidance	−0.27 [-0.43,−0.11]
Marchiol et al. (67)	AN	34	34 (100%)	25.7 (10.6)	NR	CISS avoidance distraction	EDI ED risk	Avoidance	−0.27 [-0.62, 0.08]
	BN	30	30 (100%)	24.0 (8.4)	NR	CISS avoidance distraction	EDI ED risk	Avoidance	0.02 [-0.36, 0.40]
	BED	29	29 (100%)	37.5 (12.7)	NR	CISS avoidance distraction	EDI ED risk	Avoidance	0.01 [-0.38, 0.39]
Spoor et al. (68)	AN, BN, BD, EDNOS	125	125 (100%)	29.9 ( 9.2)	NR	CISS avoidance distraction	DEBQ emotional eating	Avoidance	0.46 [0.18, 0.64]
Nagata et al. (34)	AN, BN	161	161 (100%)	22.8 (4.6)	16.5 (2.6)	CISS avoidance distraction	EDI drive for thinness	Avoidance	0.28 [0.12, 0.43]
Brytek-Matera and Schiltz (20)	AN, BN	52	52 (100%)	19.6 (2.6)	18.1 (2.5)	SVF120 Avoidance	ED pathology	Avoidance	0.35 [0.07, 0.63]
Rothschild-Yakar et al. (84)	AN	41	41 (100%)	17.6 (2.6)	18.0 (1.7)	DSI emotional cut-off	EAT total	Avoidance	0.38 [0.06, 0.69]
Tasca et al. (85)	AN, BN, EDNOS	310	310 (100%)	26.3 (8.8)	21.9 (6.2)	DSI-R emotional cut-off	EDI total	Avoidance	0.41 [0.30, 0.52]
Sheffield et al. (86)	AN, BN, BED, EDNOS	124	124 (100%)	27.6 (7.8)	21.8 (9.1)	YRAI behavioural-somatic avoidance	EDI body dissatisfaction	Avoidance	0.30 [0.12, 0.48]
Spranger et al. (87)	AN, BN, BED	19	19 (100%)	30.8 (10.0)	23.5 (6.9)	YRAI total	BITE symptoms	Avoidance	−0.22 [-0.71, 0.27]
Noetel et al. (88)	AN	60	50 (100%)	15.0 (1.2)	% EBW = 86.6 (12.39)	CET avoidance	YEDE-Q: restraint	Avoidance	0.74 [0.48, 1.00]
Sauchelli et al. (89)	AN, BN, EDNOS	157	143 (91.1%)	NR	21.5 (5.2)	CET avoidance	EDI total	Avoidance	0.34 [0.18, 0.50]
Torres et al. (55)	BED	7	7 (100%)	38.3 (9.3)	34.6 (3.9)	EPS avoidance	DEBQ total	Avoidance	0.38 [-0.60, 1.36]
Bratland-Sanda et al. (90)	AN, BN, EDNOS	38	38 (100%)	30.9 (8.9)	21.6 (3.6)	REI negative affect avoidance	EDE total	Avoidance	0.87 [0.54, 1.20]
Marzola, et al. (91)	AN	81	81 (100%)	25.3 (8.5)	15.1 (2.2)	MANQ avoidance	EDI drive for thinness	Avoidance	0.17 [-0.05, 0.39]
Meyer et al. (92)	AN	13	13 (100%)	27.8 (10.0)	16.8 (2.1)	Anagram solution task: avoidance of threat words	EDI drive for thinness	Avoidance	−0.12 [-0.74, 0.50]
	BN	37	37 (100%)	25.2 (6.8)	19.4 (1.7)	Anagram solution task: avoidance of threat words	EDI drive for thinness	Avoidance	0.13 [-0.21, 0.47]
Merwin et al. (93)	AN	6	6 (100%)	12 – 18	17.7 (1.7)	AFQ-Y	EDEQ total	Avoidance	0.85 [-0.28, 1.99]
Oldershaw et al. (72)	AN, EDNOS	40	37 (92.5%)	26.0 (8.8)	16.6 (1.3)	DTS avoidance of affect	EDEQ total	Avoidance	0.59 [0.27, 0.91]
Corstorphine et al. (71)	AN, BN, EDNOS	72	72 (100%)	24.5 (7.8)	22.7 (8.7)	DTS avoidance of affect	EDI drive for thinness	Avoidance	−0.10 [-0.34, 0.13]
Lampard et al. (94)	AN, BN, EDNOS	257	257 (100%)	26.1 (9.1)	19.8 (2.7)	DTS avoidance of affect	EDEQ total	Avoidance	0.12 [-0.002, 0.24]
Rienecke et al. (95)	AN, BN, BED, OSFED, ARFID	613	514 (83.8%)	24.5 (9.8)	NR	PCL-5 avoidance	EPSI restraint	Avoidance	0.32 [0.24, 0.40]
Baños et al. (96)	AN	66	66 (100%)	28.0 (9.0)	16.7 (1.2)	TCI harm avoidance	DEBQ restraint	Avoidance	0.27 [0.02, 0.52]
Danner et al. (97)	AN, recovered AN	45	45 (100%)	25.3 (5.0)	17.9 (1.8)	TCI harm avoidance	EDEQ total	Avoidance	0.45 [0.15, 0.75]
Díaz-Marsá et al. (98)	AN, BN	72	72 (100%)	21.5 (4.2)	NR	TCI harm avoidance	BITE total	Avoidance	0.58 [0.34, 0.81]
Duffy et al. (99)	AN	270	95.2%	28.5 (10.7)	18.7 (2.4)	TCI harm avoidance	EDI drive for thinness	Avoidance	0.28 [0.16, 0.40]
Frank et al. (100)	AN	56	56 (100%)	16.6 (2.5)	15.9 (0.9)	TCI harm avoidance	EDI drive for thinness	Avoidance	0.40 [0.13, 0.67]
Levinson et al. (101)	AN	732	NR	13 – 65	NR	TCI harm avoidance	EDI drive for thinness	Avoidance	0.23 [0.16, 0.31]
del Pino-Gutiérrez et al. (102)	BN	527	93.1%	26.0 (6.6)	NR	TCI harm avoidance	EDI total	Avoidance	0.67 [0.58, 0.75]
Paganini et al. (103)	AN, BN, OSFED	292	292 (100%)	28.1 (9.7)	24.4 (5.8)	TCI harm avoidance	EDE total	Avoidance	0.18 [0.07, 0.30]
Rotella et al. (104)	AN, BN, BED	166	166 (100%)	37.9 (14.4)	NR	TCI harm avoidance	EDEQ total	Avoidance	0.21 [0.06, 0.37]
Van Riel et al. (105)	BED, sub-threshold BED	198	198 (100%)	40.7 (12.9)	39.7 (6.2)	TCI harm avoidance	EDEQ total	Avoidance	0.20 [0.06, 0.34]
Walden et al. (50)	AN, BN, EDNOS	617	(94.6%)	28.0 (10.0)	NR	TCI harm avoidance	EDI drive for thinness	Avoidance	0.23 [0.16, 0.31]
Wierenga et al. (106)	Remitted BN	23	23 (100%)	27.2	22.0	TCI harm avoidance	EDI drive for thinness	Avoidance	0.39 [-0.05, 0.83]
Solmi et al. (107)	AN, BN, BED	2068	96.6%	28.2 (9.3)	NR	TPQ harm avoidance	EDI drive for thinness	Avoidance	0.26 [0.22, 0.30]
Marzola et al. (108)	Mixed ED	112	NR	24.8 (8.5)	15.9 (3.7)	PCT-Q harm avoidance	EDI drive for thinness	Avoidance	0.42 [0.24, 0.61]
**Rumination**									
Verschueren et al. (109 )	AN, BN, BED, EDNOS	121	121 (100%)	28.5 (9.9)	22.3 (range = 13.7 – 49.1)	DIDS ruminative exploration	EDI total	Rumination	0.20 [0.02, 0.38]
Wang et al. (110)	BED	237	167 (70.5%)	47.9 (10.0)	39.5 (5.9)	RRS brooding rumination	EDEQ total	Rumination	0.37 [0.24, 0.50]
Sagiv and Gvion (111)	AN, BN, OSFED	91	91 (100%)	24.0 (5.5)	19.4 (5.3)	RRS total	EDEQ binge eating	Rumination	0.13 [-0.08, 0.34]
Wang and Borders (112)	AN, BN, OSFED	85	87.1%	24.6 (10.0)	NR	RRS total	EDEQ total	Rumination	0.51 [0.29, 0.73]
Thew et al. (113)	Mixed ED	26	NR	> 18	NR	RRQ total	EDEQ total	Rumination	0.38 [-0.02, 0.79]
Cowdrey and Park (46)	AN	42	42 (100%)	24.0 (8.3)	19.6 (2.5)	RSS-ED brooding rumination	EDEQ total	Rumination	0.97 [0.66, 1.29]
Seidel et al. (114)	AN	37	37 (100%)	16.4 (2.3)	14.6 (1.3)	PTQ perseverative thinking	EDI total	Rumination	1.03 [0.70, 1.37]
**Suppression**									
Oldershaw et al. (72)	AN, EDNOS	40	37 (92.5%)	26.0 (8.8)	16.6 (1.3)	STSS silencing the self	EDEQ total	Suppression	0.34 [0.02, 0.67]
Overton et al. (115)	AN, BN	32	32 (100%)	28.1 (10.3)	21.5 (6.5)	YSQ emotional inhibition	EDI drive for thinness	Suppression	0.17 [-0.19, 0.54]
Torres et al. (55)	BED	7	7 (100%)	38.3 (9.3)	34.6 (3.9)	EPS suppression	DEBQ total	Suppression	0.76 [-0.22, 1.74]
Cardi et al. (116)	AN, BN, EDNOS	65	65 (100%)	25.8 (8.1)	18.9 (2.1)	Evoked facial affect	EDEQ total	Suppression	0.05 [-0.20, 0.30]
Davies et al. (117)	AN	30	30 (100%)	24.5 (range = 19.0 – 33.3)	14.6 (range = 12.9 – 15.6)	Evoked facial affect	EDEQ total	Suppression	0.46 [0.08, 0.84]
Dapelo et al. (118)	AN, BN	40	40 (100%)	27.9 (8.4)	18.9 (2.5)	Evoked facial affect	EDEQ total	Suppression	0.10 [-0.22, 0.42]
Lang et al. (119)	AN	66	66 (100%)	20.1 (7.3)	15.4 (1.4)	Evoked facial affect	EDEQ total	Suppression	0.36 [0.11, 0.61]
Leppanen et al. (120)	AN	29	29 (100%)	26.2 (7.1)	16.3 (2.1)	Evoked facial effect	EDEQ total	Suppression	−0.09 [-0.05, 0.29]
Krug et al. (121)	AN, BN, EDNOS	135	135 (100%)	25.6 (6.7)	21.9 (15.1)	STAXI-2 anger expression (out)	EDI total	Suppression	0.53 [0.36, 0.70]
Fassino et al. (122)	BED	51	51 (100%)	34.5 (8.9)	36.5 (6.3)	STAXI-2 anger expression (out)	EDI drive for thinness	Suppression	0.22 [-0.06, 0.51]
Danner et al. (75)	AN, BN, BED, EDNOS	123	123 (100%)	28.6 (8.4)	23.8 (3.3)	ERQ suppression	EDDS total	Suppression	0.08 [-0.10, 0.26]
Davies et al. (76)	AN, BN	103	103 (100%)	26.4 (8.5)	18.1 (4.1)	ERQ suppression	EDEQ total	Suppression	0.47 [0.28, 0.67]
Rothschild-Yakar et al. (77)	AN, BN	25	25 (100%)	17.2 (2.8)	20.4 (4.0)	ERQ suppression	EAT total	Suppression	0.19 [-0.23, 0.61]
Svaldi et al. (41)	AN, BN, BED	63	63 (100%)	30.7 (8.6)	25.4 (4.3)	ERQ suppression	EDEQ total	Suppression	0.51 [0.26, 0.76]

Thirty-eight studies examined associations between emotional awareness and ED symptomatology. Nine different measures were used to assess awareness of emotions (Table 1), eight of which were self-report questionnaire measures. The most commonly used measure was the DERS lack of emotional awareness subscale (N = 24). One of the measures used, the Levels of Emotional Awareness, is a performance based measure assessing participants' awareness of their own and other's emotions. Although this review builds on the previous meta-analytic review by Prefit et al. (12) we did not include emotional clarity as a separate category. We found that very few measures assessed clarity and those that did used methods that were very similar to ones used to evaluate emotional awareness. For instance the Attention and Clarity of One's Feelings and the Feelings of Others (ACF) questionnaire is described as measuring emotional self-awareness and includes questions about a persons awareness of and clarity about their own emotions. Therefore, for the sake of simplicity, emotional awareness and clarity were combined and in the case of the DERS questionnaire, which assesses both separately, only the lack of emotional awareness subscale was used. Studies assessing mindfulness were also combined with those investigating emotional awareness and clarity as the measures used were deemed to be very similar, examining similar underlying construct of paying attention to one's feelings and knowing what are the emotions one is feeling. As above, the correlations that included measures of emotional awareness, were reversed, while those that included measures of lack of awareness or difficulties in emotional awareness were kept as is. Therefore, this review ended up assessing associations between lack of emotional awareness and ED symptomatology.

Thirty-four studies examined associations between the impact of emotions on participant's problem solving abilities and ED symptomatology (Table 1). Six different measures were used to assess problem solving, four of which were self-report questionnaires and the most commonly used measure of problem solving was the DERS difficulties with goal directed behaviors subscale (*N* = 24). One of the measures, the Means-Ends Problem-Solving Test [MEPS; (124)], was a performance-based measure used to evaluate the effectiveness of participants problem solving strategies. In this task participants are given the beginning and end of four different scenarios and they are then asked to provide the middle part connecting the beginning and ending. In all but two of the measures a higher score indicated better problem solving and thus, the correlation coefficients were reversed for the meta-analysis to assess association between problem solving difficulties and ED symptomatology. The Social Problem-Solving Inventory-Revised [SPSI-R; (125)] includes the negative problem orientation subscale and the DERS includes difficulties with goal directed behaviors subscale, both of which assess negative approach that prevents effective problem solving. This subscale was used in the present review.

Eight studies examined associations between cognitive reappraisal of emotions and ED symptomatology (Table 1). Two different measures were used to assess cognitive reappraisal, both of which were self-report questionnaires and the most commonly used measure was the Emotion Regulation Questionnaire [ERQ; (126)] reappraisal subscale (*N* = 5). In both questionnaires, higher scores indicated greater use of cognitive reappraisal and thus, the correlation coefficients were reversed to assess associations between lack of cognitive reappraisal and ED symptomatology.

#### Maladaptive Emotion Regulation Measures

Forty-seven studies investigated associations between emotional avoidance and ED symptomatology (Table 1). Twenty-one different measures were used to assess emotional avoidance, 16 of which were self-report questionnaires; the most commonly used measure was the Temperament and Character Inventory [TCI; (127)] harm avoidance subscale (*N* = 13). One of the measures, the Anagram solution task, used a behavioral measure of avoidance of threatening words. In this task, participants were given a set of anagrams to solve and the time taken to reach the correct solution was used to measure avoidance. In all measures, higher scores indicated more avoidance.

Seven studies examined associations between rumination and ED symptomatology (Table 1). Four different measures were used to assess rumination, all of which were self-report questionnaires. The most commonly used measure to assess rumination was the Ruminative Response Scale [RSS; (128)] (*N* = 5). One of the questionnaires used was adapted from the RSS to assess ED specific rumination (46). In all measures used, higher scores indicated more rumination.

Thirteen studies investigated association between emotion suppression and ED symptomatology (Table 1). Six different measures were used to assess emotion suppression, five of which were self-report questionnaires and the most commonly used measure was the only task-based assessment, which examined evoked facial affect (*N* = 5). In the Evoked facial affect task, participants were presented with emotionally provoking stimuli and their evoked facial expressions were analyzed. In this task, higher scores indicated more emotion expression and less suppression, and thus, the correlation coefficients were reversed to reflect reverse scoring of the task. In all self-report measures, higher scores indicated more emotion suppression.

### Quality Assessment

Quality assessment was conducted by J.L. and D.B. using the NIH Quality Assessment Tool for Observational Cohort and Cross-Sectional Studies (https://www.nhlbi.nih.gov/health-topics/study-quality-assessment-tools) to investigate study level bias and the results are shown in Supplementary Data. Question relating to exposures were not considered as they were not relevant for the purpose of the present review, which focused on correlations prior to any potential interventions or exposures. Inter-rater reliability was good [intra-class correlation coefficient = 0.94, 95% CI (0.93, 0.96)]. Any disagreements were resolved through group discussion. All included studies were deemed to be high enough quality to be included in the meta-analysis and the sum of the quality scores were included in a meta-regression to examine whether the study quality could explain any potential network inconsistency.

### Statistical Analysis

We conducted a Bayesian network meta-analysis to examine which aspects of emotion regulation were most closely associated with ED symptomatology. The meta-analysis was conducted in R (129) using the packages *gemtc* (130) and *metafor* (131). First, all relevant correlation coefficients of the association between an aspect of emotion regulation and ED symptomatology were extracted from the included studies. The Pearson's *r* coefficients were then adjusted using sample-size weights to approximate population correlation and Fisher's r-to-z transformed was conducted using the function *escalc* from the package *metafor*. We converted the correlation coefficients to z-scores for the purposes of the network meta-analysis as z-scores are not bounded and come from a normal distribution. We then calculated standard error for each study from the sample size adjusted variance using the following steps


$$\begin{array}{l} \sigma =\sqrt{v}\\ SE=\frac{\sigma }{\sqrt{n}} \end{array}$$

where *v* is the estimated, sample size adjusted variance, σ is the standard deviation, and *n* is the sample size. The sample size adjusted correlation coefficients and standard errors were then taken forward to conduct the network meta-analysis.

First, we generated an initial network of the data where edge thickness represents the number of studies that reported that correlation. The generated network object was then taken forward to specify and compile the random effects model with four Markov chains and a normal likelihood function with an identity link. Next, a Markov Chain Monte Carlo (MCMC) simulation was conducted to estimate the posterior probabilities. To ensure convergence we specified 1,00,000 iterations with 5,000 burn-in iterations. The Gelman-Rubin plots are presented in Supplementary Figure 1. The parameter estimation is conducted by utilizing Just Another Gibbs Sampler (JAGS). We then generated rank probability and forest plots to visualize the direct comparisons between different aspects of emotion regulation and to examine which aspect was the most closely associated with ED symptomatology. Additionally, we also calculated Surface Under the Cumulative Ranking (SUCRA) score to evaluate which aspect of emotion regulation is most relevant in terms of ED symptomatology and might serve a useful target for interventions.

Network inconsistency was examined using a network splitting method and node comparisons with Bayesian *p* < 0.05 were considered to be indicating inconsistency in the network. As we included studies with adolescent and adult participants of any gender who had any ED diagnosis, meta-regressions were conducted to explore if any possible network inconsistency could be explained by between-study heterogeneity in study quality score, age, BMI, and whether the studies had female only or mixed samples. The meta-regressions were conducted by taking the above steps with a specified regressor.

This review has been registered on PROSPERO (ID: CRD42021249996) and the code and data used to conduct the meta-analysis is available on the Open Science Framework online repository (https://osf.io/gz3kt/?view_only=6fe361c208e04817b820c1f3fb2fd2b5).

## Results

### Study Characteristics

The characteristics of each study included in the review are summarized in Table 1. Altogether, data from 19,734 participants were included in the meta-analysis, with average study sample size of 181 (range = 6–2,405). Majority, 72, of the studies included only female participants and three studies did not report the participants' gender. Additionally, most studies included a mixed ED sample and assessed emotion regulation across ED diagnoses while 50 studies examined emotion regulation within one diagnostic group. The mean age across studies was 27.7 (range = 15.0–49.3); most studies included adults and only eight studies included a sample of adolescents with sample mean age below 18. The mean BMI across studies was 22.25 (range = 14.6–39.7), with most studies including people who were of healthy weight, and 29 studies including underweight participants with sample mean BMI below 18.5 and 15 studies including overweight participants with sample mean BMI above 25.

### Network Characteristics

**Figure 2:**
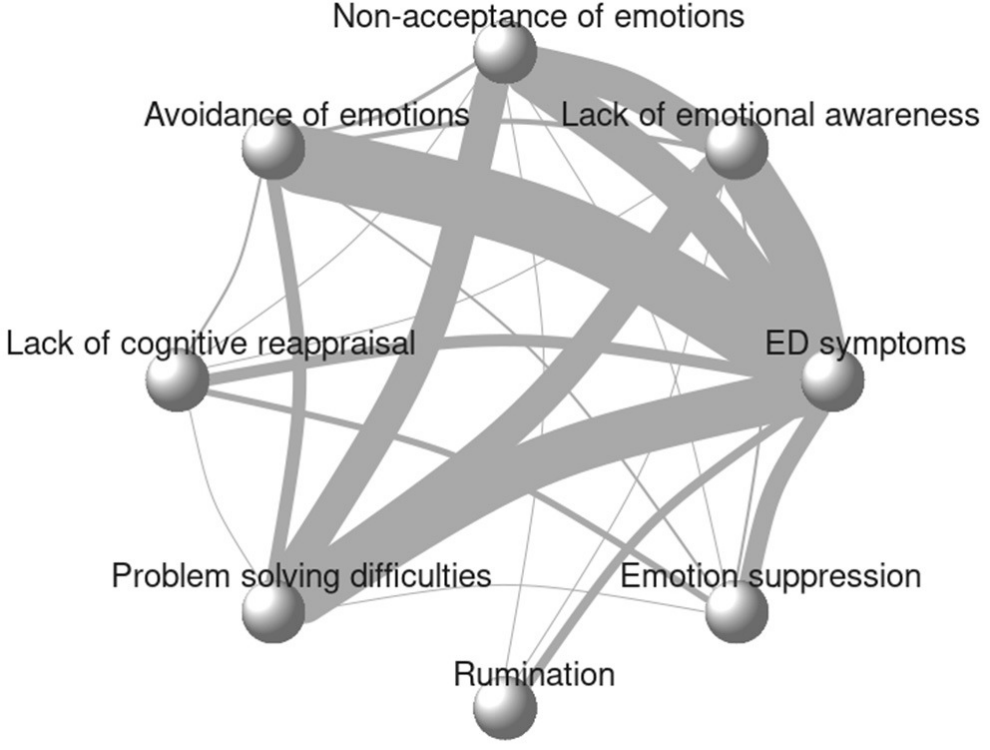
Initial network plot. Line thickness represents the number of studies reporting the association.

The initial network graph, in Figure 2, shows the eligible comparisons to identify emotion regulation strategy most strongly linked to ED symptomatology. The thickness of the edges indicates the number of studies that evaluated a given association or comparison. In the present review most of the studies (*N* = 50) examined the association between avoidance and ED symptomatology. Across the 104 included studies, 294 correlations contributed to the network. All emotion regulation strategies were compared with at least one other emotion regulation method in addition to the association with ED symptoms resulting in a well-connected network, which is more likely to produce reliable results (132).

### Forest Plot

**Figure 3:**
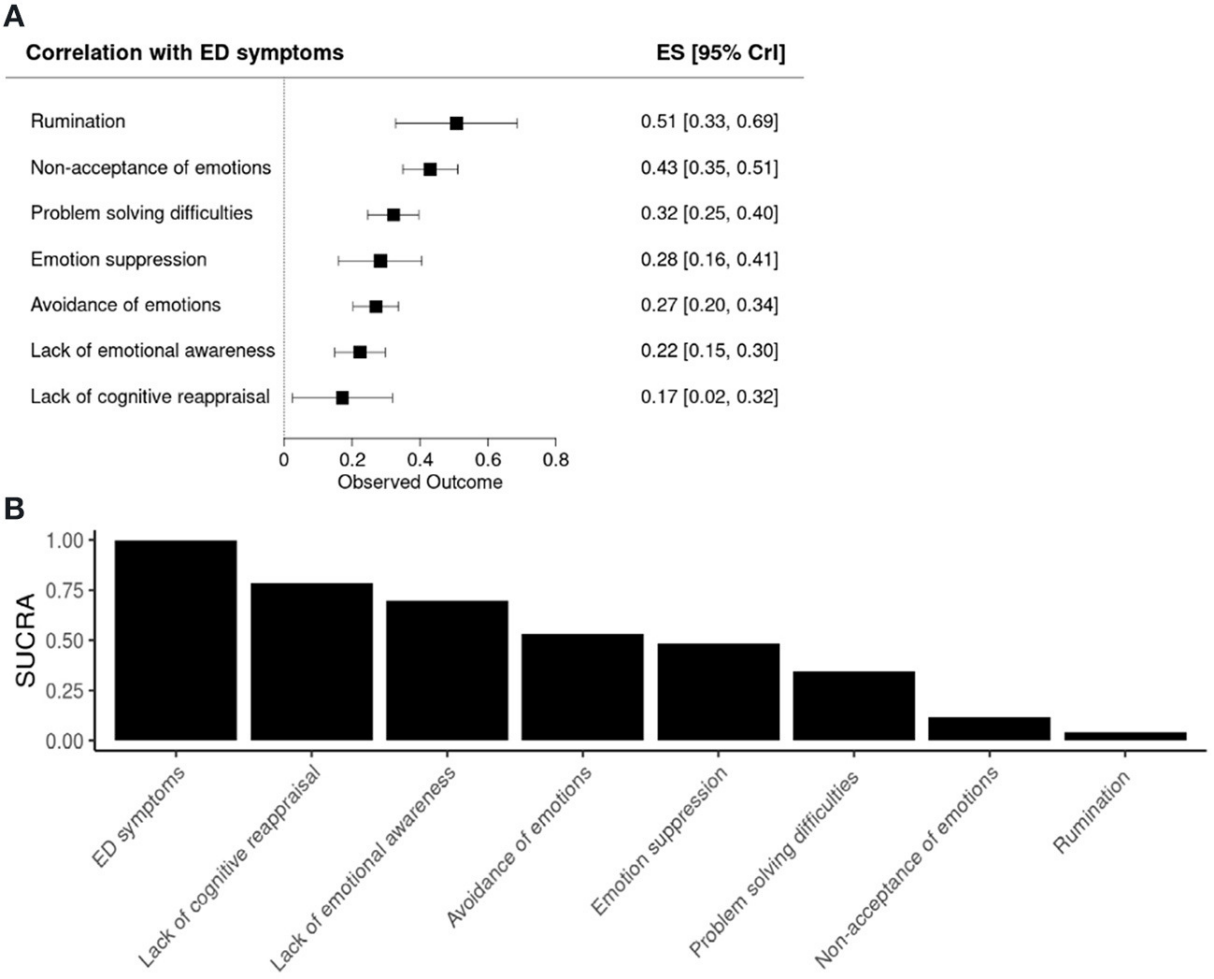
Forest and SUCRA plots. **(A)** Forest plot showing the associations between the emotion regulation strategies and ED symptoms. **(B)** Bar plot showing the surface under the cumulative ranking (SUCRA) for each emotion regulation method. ES, effect size; CrI, Credible Interval.

The forest and SUCRA plots from the Bayesian random effects network meta-analysis are shown in Figure 3. Each emotion regulation strategy represents a unit of analysis and is thus presented on the rows. Larger positive effect sizes indicate a stronger association between a given emotion regulations strategy and ED symptomatology. The meta-analysis showed that the emotion regulation strategies vary in their relationship with ED symptomatology, with two methods showing the strongest association: rumination [ES = 0.51, 95% CrI (0.33, 0.69)] and non-acceptance of emotions [ES = 0.43, 95% CrI (0.35, 0.51)]. Lack of cognitive reappraisal had the weakest association with ED psychopathology [ES = 0.17, 95% CrI (0.02, 0.34)].

### Meta-Regressions

Network inconsistency was evaluated using the node-split method (Supplementary Figure 2). Although there was no evidence of significant inconsistency, the comparisons between problem solving difficulties and lack of emotional awareness (*p* = 0.076) and rumination and non-acceptance of emotions (*p* = 0.084) approached significance. Therefore, three meta-regressions were conducted to examine whether these slight inconsistencies could be explained between-study differences in study quality score, BMI, age, or whether the study included only female participants or a mixed sample of participants. The meta-regressions revealed a significant effect of BMI [*b* = −0.09 CrI (−0.18, −0.01)] such that the associations between different emotion regulation strategies and ED psychopathology were weaker among those with low BMI. There was no significant effect of study quality [*b* = −0.01, CrI (−0.09, 0.12)], age (*b* = −0.04, CrI (−0.14, 0.06)] or whether the studies included of only female or mixed participants [*b* = 0.02, CrI (−0.12, 0.32)].

## Discussion

The aim of the present review was to examine associations between various adaptive and maladaptive emotion regulation strategies and ED psychopathology trans-diagnostically to identify strategies that were most closely linked with psychopathology. We used network meta-analysis approach with a well-connected network, which identified rumination and non-acceptance of emotions to be most closely associated with ED symptomatology. Difficulties in cognitive reappraisal was found to be the least connected with ED symptomatology of all emotion regulation strategies examined in this review. There was some evidence of network inconsistency which approached significance and two meta-regressions were performed to examine if this could be explained by the between-study variability in BMI or age. The meta-regressions were non-significant suggesting the findings were consistent across age groups and BMI.

The present review adds to the steady accumulation of evidence highlighting the relationship between maladaptive emotion regulation based on rumination and ED symptomatology (133, 134). Over the recent years, studies using ecological momentary assessments (EMAs) have reported that rumination and repetitive negative thinking predicts engagement in ED behavior, including body checking and binge eating (135–137). One EMA study also reported that excessive rumination also predicted higher levels of ED psychopathology at a 1 month follow-up assessment (135). Moreover, it has been suggested that some ED symptoms, such as excessive focus on food, eating, and body weight and shape, are forms of illness specific rumination (138). Indeed, a longitudinal study using EMA and biological assessments found that food-related rumination was linked to BMI status and leptin levels among people in treatment for anorexia nervosa (139). This suggest that at least certain aspects of illness-specific rumination may be linked to under-nutrition and physiological signaling, explaining why weight restoration can have a positive impact on this type of rumination. Overall, it appears that rumination, whether general repetitive negative thinking or illness-specific, is a key characteristic of the acute stage of an ED.

It is important to note that most of the studies included in the present review used the RRS questionnaire, which assesses brooding, depressive rumination, and reflection. Therefore, it is possible that the observed strong association between rumination and ED psychopathology may be partially explained by known links between depression and ED symptoms (140, 141). Indeed, one study reported that although food-related rumination was linked to stage of illness and under-nutrition, other aspects of illness-specific rumination including, body weight and shape, were more associated with negative mood than not ED symptoms (139). Additionally, an experimental study has reported that induction of body shape related rumination had a direct negative impact on mood but not on ED cognitions among people with BED (142). Another interpretation is that the above findings may reflect the mechanism through which rumination influences EDs: due to its repetitive nature of rumination forms a habitual relationship with depressive mood which in turn fuels ED symptomatology (143). In support of this hypothesis, one longitudinal EMA study documented a bidirectional mediation between rumination, negative mood, and ED symptoms (136). The authors found that rumination mediated the association between low mood and ED symptomatology and low mood in turn mediated the association between rumination and ED symptomatology. Taken together these findings highlight the need to further examine the underlying processes through which rumination impacts EDs and how rumination may impact illness progression and recovery.

Difficulties in adaptive emotion regulation, specifically with accepting emotions, was another domain that was highly associated with ED psychopathology in the present review. This is in line with findings from the general population showing that reduced use of adaptive emotion regulation strategies, such as acceptance, and increased reliance on maladaptive methods, including suppression and avoidance, are associated with daily habit of food restriction (144). Similarly, people who report objective binge eating episodes and loss of control over eating also report more difficulties accepting emotions than those who do not engage in disorder eating behaviors (145). Additionally, a recent longitudinal study reported that a poor response to CBT-based ED treatment was associated with lack of change in the patients' self-reported ability to accept emotions and limited access to other effective emotion regulation strategies (146). Moreover, another interventional study found that irrespective of treatment condition, changes in acceptance of emotions were associated with greater improvements in ED-related quality of life post-intervention (27). These findings have led some authors to suggest that emotion regulation in general and acceptance of unwanted emotions in particular should be added to current standard ED treatments (146, 147). While it seems that acceptance of emotions may have a strong role in disordered eating and the progression of EDs, further investigation of the underlying mechanism would be of interest to aid the development of evidence-based interventions.

Interestingly, difficulties accepting emotions has also been proposed to be linked to excessive reliance on other maladaptive emotion regulation strategies, including rumination, avoidance, and suppression (148, 149). If unwanted emotions are deemed unacceptable, a person might put great effort in avoiding situations that give rise to such emotions or, if the emotions are already present, engage in suppression in an attempt to manage the unwanted emotions. Findings from a review of experimental and self-report studies support this notion reporting links between suppression and non-acceptance of emotion among people with AN and BN (150). Difficulties accepting and general dislike of emotions have been linked to greater general tendency to engage in rumination as well as worry and low mood (151). Interestingly, difficulties in emotion regulation, including non-acceptance of emotions, have been found to mediate the association between experiential avoidance and ED psychopathology among people with a range of ED diagnoses (44). Similar findings have been reported in the general population with reduced access to adaptive emotion regulation strategies, including acceptance of emotions and reappraisal, and increased reliance on maladaptive strategies based on suppression being associated with greater tendency to engage in ED-related behaviors (144). Thus, further investigation of the mechanisms that might underlie this connection as well as examination of the impact of acceptance-focused interventions on the use of other emotion regulation strategies among people with EDs may be of interest.

Interestingly, our meta-regression also found a significant effect of BMI, such that the associations were generally weaker among those with lower BMI. This is somewhat in contrast with previous reviews suggesting that there are no significant differences in the associations between emotion regulation methods and ED psychopathology between different ED diagnostic groups characterized by low and high BMI (12, 13). However, some studies have reported that starvation impacts emotion regulation in such a way that those with very low BMI in the acute stage of AN report fewer difficulties (152). Indeed, it has been suggested that self-starvation itself works as an emotion regulation strategy which reduces both the internal experience and external expression of emotions through (153). By suppressing physiological responses and arousal, starvation can help the person escape and avoid unwanted emotions (153, 154). If no alternative methods are available, the person may over time become reliant on starvation as their sole emotion regulation strategy due to its numbing effect (155, 156). This mechanism could be one of the factors contributing to the present finding and it further highlights the complex relationship between emotion regulation and EDs.

### Clinical Implications

Over the recent years, several reviews have recommended the use of interventions aimed at reducing rumination and repetitive negative thinking, Metacognitive Therapy (MCT) and Rumination-Focused Cognitive Behavioral Therapy (RFCBT), such as in the treatment of EDs (10, 12, 133, 134). MCT and RFCBT have been successfully used to treat anxiety and depression among other psychiatric disorders, and there is some evidence suggesting that rumination-focused treatments may help reduce the risk of relapse in depression (157–159). Furthermore, one of the meta-analytic reviews found a very high correlation between rumination and ED symptomatology among the general population, which led the authors to suggest that rumination may be a useful target for interventions aiming to prevent EDs and other forms of disordered eating (133). To date, very few studies examining the impact of interventional designed to target rumination in EDs have been conducted. To our knowledge only one case series has explored the effects of MCT on binge eating behavior among three people with BED (160). MCT aims to alleviate repetitive negative cognitions by increasing awareness and mindfulness, and modifying the metacognitions that underlie maladaptive behaviors, such as binge eating (161, 162). The Robertson and Strodl (160) found that MCT intervention significantly reduced binge eating frequency and improved cognitions related worry and rumination. There improvements were maintained at a 2-month follow-up assessment. Together, these findings highlight the need to develop new interventions or adapt existing treatments to target rumination and repetitive negative thinking in EDs.

Acceptance and mindfulness based interventions, such as Acceptance and Commitment Therapy (ACT), have been proposed to target difficulties in coping with unwanted emotions in EDs (147, 163). The purpose of ACT is to encourage people to accept and experience unwanted emotions without attempts to modify them, thus reducing avoidance and suppression of difficult emotions and embracing the use of adaptive emotion regulation strategies (163). A number of small case series have reported that ACT can be effective in reducing ED symptomatology and behaviors among people with AN and BED (164–166). Another larger longitudinal treatment study found that ACT was more effective than treatment as usual in reducing residual ED symptoms and risk or relapse following standard ED treatment and the effects were maintained at a 2-year follow-up (167). However, another interventional study found that ACT did not lead to greater improvements in ED related quality of life than treatment as usual (27). Furthermore, a systematic review examining the use of ACT to treat body image disturbance and weight dissatisfaction reported that they could not determine the effectiveness of ACT due to the poor quality of current evidence (168). These findings suggest that acceptance and mindfulness based interventions may be promising in the treatment of EDs, but more research into the mechanisms that underlie the relationship between emotion acceptance and ED psychopathology is needed to create evidence-based treatment strategies.

### Limitations

The main limitation of this review was the use of the network meta-analysis due to its use of indirect evidence, which relies on the assumption of transitivity (169). According to the transitivity assumption, a given associations is exchangeable between studies even if a given study did not assess that association. This assumption can be violated by individual differences in the samples between studies, which can be difficult to control resulting in network inconsistency. Although we did not observe significant network inconsistency, there was evidence of near significant inconsistency in two comparisons. Moreover, since we used the network approach to meta-analysis we were able to only include studies that reported correlation coefficients, which led to the exclusion of a substantial number of otherwise relevant studies. Although we attempted to contact the corresponding authors of all papers which did not report the relevant data, we were able to gain access to correlation coefficients from only 28 studies through personal correspondence. Including only a subset of the available literature to the meta-analysis may have impacted the findings.

Another limitation of this review is that majority of the studies included adult women with normal BMI. Although the meta-regressions indicated that age did not have a significant impact on the results, findings from the present review may not be fully generalizable to all age groups. Additionally, even though we did find a significant effect of BMI, we were not able to examine the impact of diagnostic group as the *gemtc* package used in the present analysis does not presently handle categorical covariates. To truly examine the potential transdiagnostic nature of emotion regulation difficulties in EDs, a direct comparison of different diagnostic groups would be needed rather than solely focusing on BMI. Furthermore, the findings may not be fully generalisable to other genders. Gender identity can impact a person's experiences with the world around them and thus influence their emotion regulation habits (170, 171). Thus, further exploration of the impact of gender and particularly minority gender identity on emotion regulation in EDs is needed. Finally, it is also important to note that we did not have equal number of studies examining each association. Indeed, only eight studies reported correlations involving difficulties with cognitive reappraisal and this method was found to be most weakly associated with ED psychopathology with largest between study variance. It is possible that this finding may have been impacted by the small number of studies included. However, only seven studies reposted correlations involving rumination and this maladaptive strategy was found to be most strongly associated with ED psychopathology. Still, equal and large number of studies examining each association would enable us to draw stronger conclusions.

## Conclusions

This review aimed to build on previous work by conducting a trans-diagnostic network meta-analysis to identify emotion regulation strategies most closely associated with psychopathology among those with ED. The meta-analysis revealed that rumination and difficulties accepting emotions were most closely associated with ED symptoms, while the weakest association was between difficulties with cognitive reappraisal and ED symptoms. The meta-regressions showed that BMI had significant impact such that the associations between various emotion regulation strategies and ED psychopathology were weaker among those with low BMI. The present findings add to the steady accumulation of evidence highlighting the relationship between ED psychopathology and reliance on maladaptive emotion regulation strategies based on rumination and non-acceptance of emotions. Together with previous longitudinal observational studies and ecological momentary assessments, these findings suggest that these two maladaptive strategies may have a key role in maintaining and perpetuating ED. The meta-regression finding also emphasizes the complex relationship between ED symptoms and emotion regulation. It is possible that some people with ED may use starvation and malnutrition to escape and avoid unwanted emotions. Thus, there is pressing need to explore and develop interventions targeting emotion regulation difficulties in ED with particular focus on rumination and non-acceptance of emotions.

## Data Availability Statement

Publicly available datasets were analyzed in this study. This data can be found here: https://osf.io/gz3kt/?view_only=6fe361c208e04817b820c1f3fb2fd2b5.

## Author Contributions

JL: conceptualization, methodology, investigation, formal analysis, visualization, writing—original draft, and writing—review and editing. DB and HM: methodology, investigation, validation, and writing—review and editing. SW and KT: conceptualization, writing—review and editing, and supervision. All authors contributed to the article and approved the submitted version.

## Funding

This research was funded in whole, or in part, by the Wellcome Trust [213578/Z/18/Z]. For the purpose of open access, the author has applied a CC BY public copyright licence to any Author Accepted Manuscript version arising from this submission. The research was further supported by MRC-MRF Fund [MR/R004595/1]. The funding bodies did not play an active role in the design of this study, nor in data collection or analysis, nor in writing the manuscript.

## Conflict of Interest

The authors declare that the research was conducted in the absence of any commercial or financial relationships that could be construed as a potential conflict of interest.

## Publisher's Note

All claims expressed in this article are solely those of the authors and do not necessarily represent those of their affiliated organizations, or those of the publisher, the editors and the reviewers. Any product that may be evaluated in this article, or claim that may be made by its manufacturer, is not guaranteed or endorsed by the publisher.
