# Supplementary figures and images for: The Role of Emotion Regulation in Eating Disorders: A Network Meta-Analysis Approach
Source: Front Psychiatry. 2022 Feb 23;13:793094. doi: 10.3389/fpsyt.2022.793094 (PMC8904925; doi:10.3389/fpsyt.2022.793094)

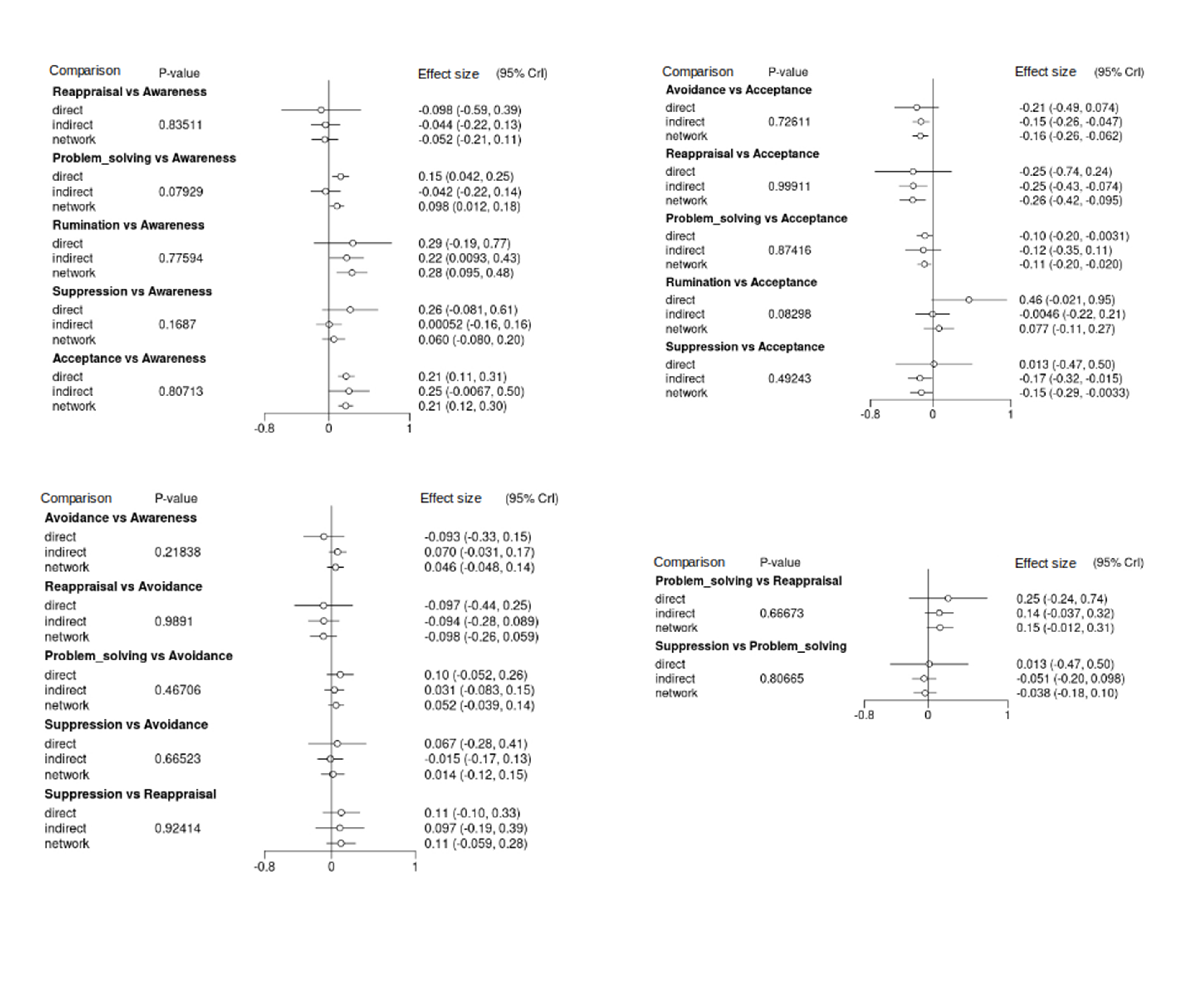

Supplement: Supplementary file 2 [file Image_1.TIF]

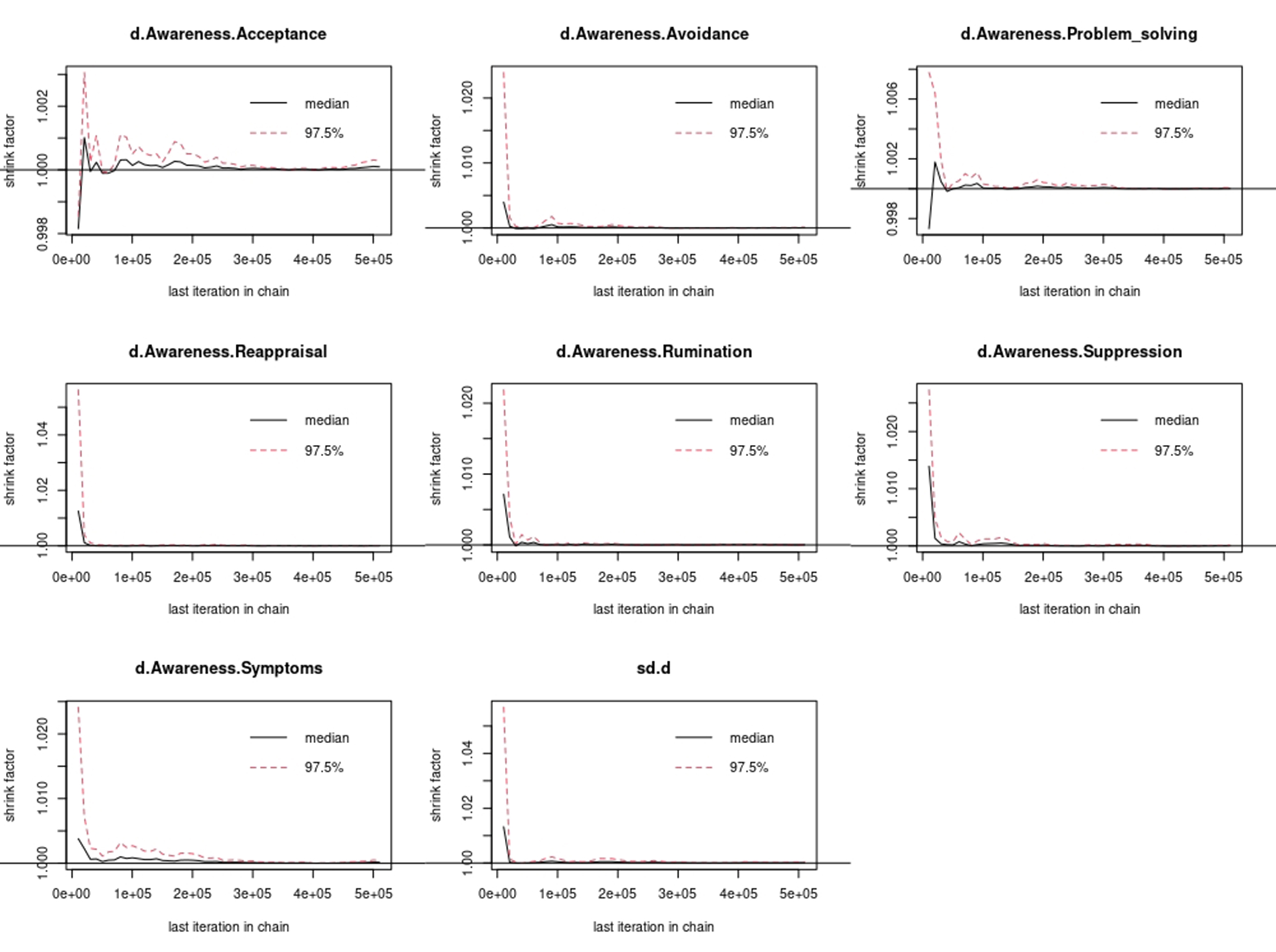

Supplement: Supplementary file 3 [file Image_2.JPEG]
